# Crystal structure of poly[[μ_3_-(*S*)-2-amino-3-hydroxy­propano­ato]-*cis*-di-μ-chlorido-caesium­palladium(II)]

**DOI:** 10.1107/S2056989017016164

**Published:** 2017-11-21

**Authors:** Mohan Madhav Bhadbhade, Alexander J. Charlson

**Affiliations:** a Mark Wainwright Analytical Centre, University of New South Wales, Sydney, NSW 2052, Australia; b Department of Chemistry and Biomolecular Sciences, Macquarie University, North Ryde, NSW 2109, Australia

**Keywords:** palladium–amino acid complex, l-serine, single-crystal X-ray structure, palladium complex

## Abstract

The structure of the title compound, [CsPd(C_3_H_6_NO_3_)Cl_2_]_*n*_, previously shown to have anti­cancer activity in rodent test systems and recently found to have anti­fungal activity, has been determined. The Pd centre is in a square-planar coordination environment with two chlorine atoms in *cis* positions and the remaining two coordination sites being coordinated by N and O atoms from deprotonated l-serine. Each of the Cs cations shows ninefold coordination with six chlorine and three O atoms resulting in a coordination environment that is similar to the well known Cs_2_SO_4_ structure. X-ray crystal structures of only three di­chlorido­palladium(II)–amino acid complexes have been determined so far and the present paper describes one of those.

## Abstract 2

This compound was previously shown to have anti­cancer activity in rodent test systems and recently found to have anti­fungal activity. The Pd centre is in a square-planar coordination environment with two chlorine atoms in *cis* positions and the remaining two coordination sites being coordinated by N and O atoms from deprotonated l-serine. Each of the Cs cations shows ninefold coordination with six chlorine and three O atoms resulting in a coordination environment that is similar to the well known Cs_2_SO_4_ structure.

## Chemical context   

The X-ray crystal structure of potassium-l-alaninato-di­chloro­platinate(II) has been published (Schiesser *et al.*, 2012 ▸). Two complexes of l-serine with palladium(II), bis(l-serinato) palladium(II) and caesium *cis*-di­chloro-l-serinato palladium(II), were synthesized (Charlson *et al.*, 1981 ▸) and an X-ray crystal structure determination of bis (l-serinato) palladium(II) has been performed (Vagg, 1979 ▸). Previously it was shown that caesium *cis*-di­chloro-l-serinato palladium(II) produced filamentous growth in *Escherichia coli (E.coli)* bacteria (Charlson *et al.*, 1981 ▸), markedly modified the inter­ior of *E.coli* bacteria cells (McArdle *et al.*, 1984 ▸), increased the lifespan of solid murine tumors Ca-755 and RShM-5 (Treschalina *et al.*, 1994 ▸) and had radio-modifying properties (Treshalina *et al.*, 1995 ▸). Recently it was found that caesium *cis*-di­chloro-serinato palladium(II) had anti­fungal activity in the *Candida albicans* and *Cryptococcus neoformans* test-systems and was non-cytotoxic against human kidney cells at the dose levels used (Elliott, 2016 ▸). The anti­microbial screening was performed by CO–ADD (The Community for Anti­microbial Drug Discovery) funded by the Welcome Trust (UK) and the University of Queensland (Australia). In the publication describing the synthesis of caesium *cis*-di­chloro-l-serinato palladium(II), the empirical formula of the compound was deduced on the basis of the percentages of carbon, hydrogen, chlorine and nitro­gen that were obtained by micro analysis (Charlson *et al.*, 1981 ▸) The present X-ray crystal structure was performed in order to establish the mol­ecular and structural formulae of caesium *cis*-di­chloro-l-serinato palladium(II).
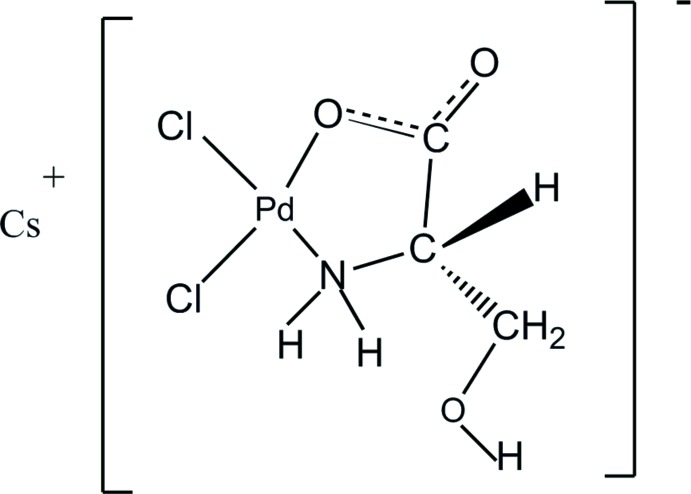


## Structural commentary   

**Figure 1:**
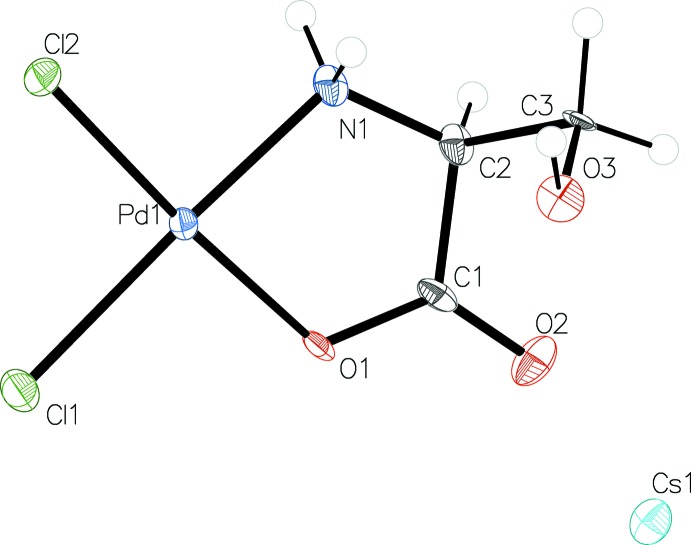
*ORTEP* representation of the asymmetric unit showing atom labelling (ellipsoids drawn at 50% probabilities).

**Figure 2:**
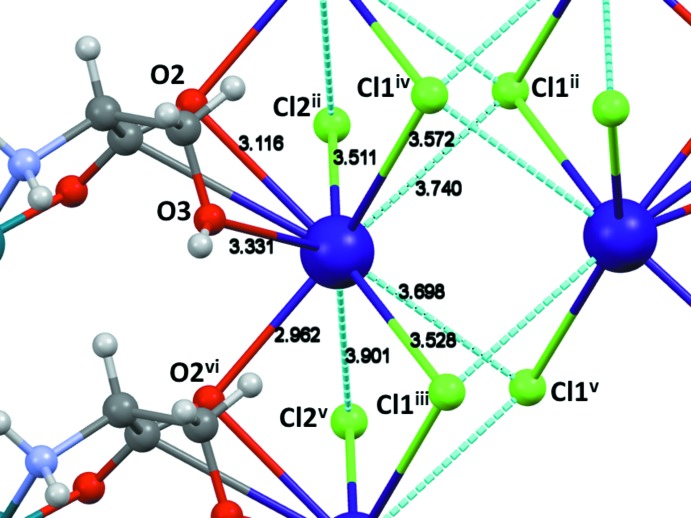
Caesium coordination and packing of cations and anions in the unit cell.

The palladium(II) serine complex ion shows a square-planar coordination of palladium with the two chloro ligands being in *cis* positions relative to each other and the remaining two coordination sites being coordinated by the nitro­gen atom (N1) and one of the carboxyl­ato oxygen atoms (O1) of the deprotonated amino acid l-serine. The view of the asymmetric unit is given in Fig. 1 ▸ and the ninefold coordination (three oxygen and six chlorine atoms) of caesium is shown in Fig. 2 ▸. A summary of significant bond distances is given in Table 1 ▸. The two Pd—Cl bonds are of slightly different bond length. The longer bond [Pd1—Cl1 = 2.305 (4) A] is *trans* to nitro­gen and the shorter one [Pd1—Cl2 = 2.287 (4) A] is *trans* to the oxygen atom. The same behaviour was observed in the structure of barium di­chloro­(glycinato) palladium(II)·2H_2_O (Baidina *et al.*, 1980*a* ▸). The five membered ring Pd1–O1–C1–C2–N1 is planar with the hy­droxy­methyl substituent in a *gauche–gauche* orientation that is very similar with the conformation of one of the ligands in the structure of bis­(l-serinato) palladium(II) (Vagg, 1979 ▸).

## Supra­molecular features   

**Figure 3:**
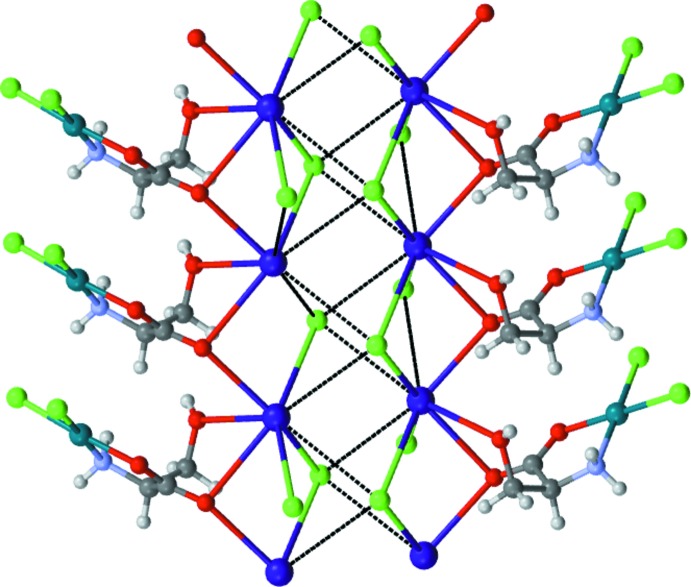
The cation and anion assembly viewed along the twofold axis (the *c* axis).

**Figure 4:**
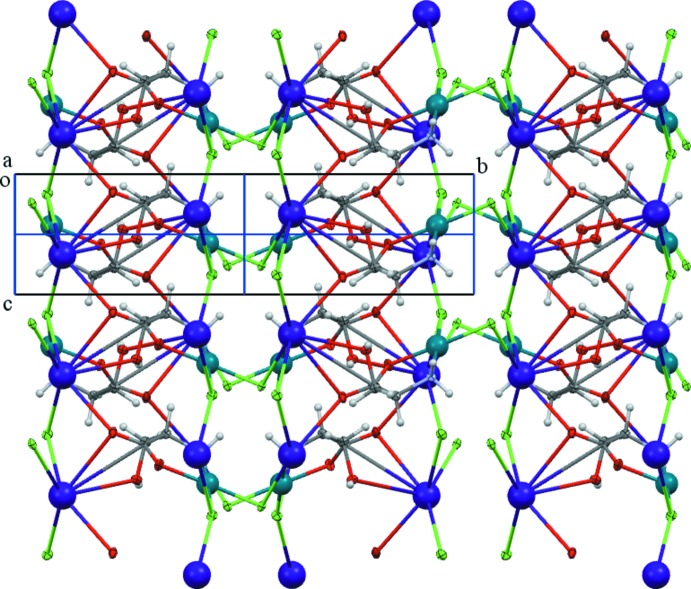
Chains of complex anions related by a 2_1_ screw axis along the *a* axis linking double rows of caesium cations (blue spheres).

The cation and anion assembly, viewed along the twofold axis (the *c* axis) is shown in Fig. 3 ▸. Chains of complex anions related by a 2_1_ screw axis along the *b* axis link double rows of caesium cations (Fig. 4 ▸). The caesium ions are bridged by chlorine atoms along and across the rows. The successful crystallization with larger Cs ions, which failed with smaller K ions, can be rationalized with this lattice arrangement. Larger cations with higher coordination capability can engage four mol­ecules of complex anions acting as a nucleator in forming the lattice much better compared to smaller cations such as Na or Li.

**Figure 5:**
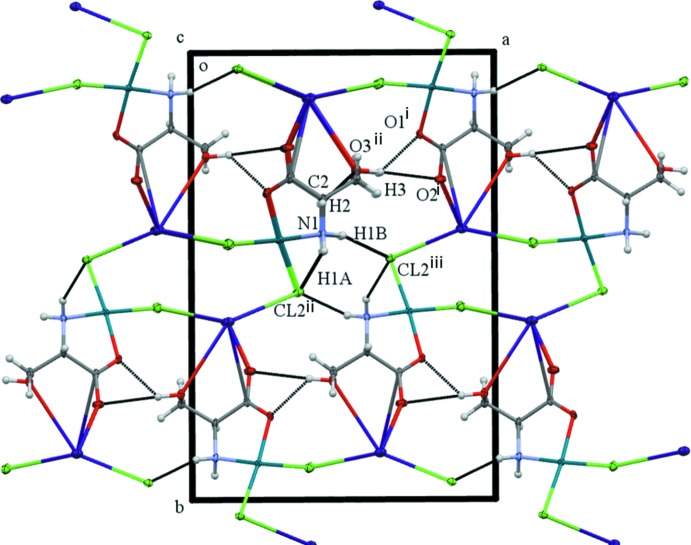
A view of the packing illustrating the hydrogen bonding (dashed lines; see Table 2 ▸).

In the crystal, extensive O—H⋯O, N—H⋯Cl and C—H⋯O hydrogen bonds (Table 2 ▸, Fig. 5 ▸) link the molecules, forming a two-dimensional network parallel to (010).

## Database survey   

The planarity of the chelate ring *M*–N–CH(*R*)–C–O is thought to be a relevant structural parameter in correlating biological activities and was examined in the structures of Pd (36 hits) and Pt (49 hits) complexes from the Cambridge Structural Database (CSD; Groom *et al.* 2016 ▸) using *CONQUEST* (Version 1.19; Bruno *et al.*, 2002 ▸). However, there are very few structure determinations of Pd or Pt complexes with amino acids as the organic ligands, only five having been reported for Pt and three for Pd. Table 3 ▸ details these structures and their chelate ring geometry parameters in relation to their planarity. It appears from Table 3 ▸ that the planarity of the five-membered ring is dependent on the hybridization state of the carboxyl­ate moiety after it has coordinated to the metal ion. Thus longer C—O bonds (associated with shorter exocyclic ones) give rise to larger O—C—C—N torsion angles (and non-planarity), whereas more equal C—O bonds form planar five-membered rings. For example, structure ACEMEC (Schiesser *et al.*, 2012 ▸) has the highest torsion angle (25.85°) accompanied by a quite long C—O bond length (1.304 Å) whereas structure BAGLPD (Baidina *et al.*, 1980*a* ▸) shows the smallest torsion angle (5.36°) together with a slightly shorter C—O bond length (1.284 Å). Irrespective of its causes (electronic factors during the complex formation), this parameter could be an important feature while modelling the inter­action of the complexes with DNA for biological activities.

## Biological considerations   

Caesium *cis*-di­chloro-l-serinato platinum(II) has been shown to increase the lifespan of P-388 leukemic mice. It also has anti-tumor activity in the MBG5 Supernal Capsule MX-1 mammary carcinoma xenograph mouse test-system (Charlson & Shorland, 1984 ▸). An X-ray crystal structure determination of caesium *cis*-di­chloro-l-serinato platinum(II) has not been performed. Since caesium *cis*-di­chloro-l-serinato platinum(II) and caesium *cis*-di­chloro-l-serinato palladium(II) both show anti­cancer activity in mouse test-systems, it may be anti­cipated that the platinum(II) complex also has a planar five-membered ring system. Recently, there has been a report on the structure of potassium (2-amino-3-hy­droxy­propano­ato)di­chloro­platinum(II) (Fabbiani *et al.*, 2015 ▸) in which one mol­ecule has a planar ring. Potassium *cis*-di­chloro-glycinato platinum(II) has also been shown to increase the lifespan of P-388 leukemic mice (Charlson & Shorland, 1984 ▸). Therefore the hydroxyl group in caesium *cis*-di­chloro-l-serinato platinum(II) plays little or no part in the anti-tumor activity shown by this complex. In the publication by Schiesser *et al.* (2012 ▸), the authors mentioned that some platinum(II) complexes with amino acid ligands showed moderate cytotoxicity toward tumor cells. However, they did not mention whether potassium-l-alaninato-di­chloro platinum(II) has been tested or not in any of the rodent test-systems. It should also be mentioned that potassium *cis*-di­chloro­glycinato platinum(II) and caesium *cis*-di­chloro-l-serinato platinum(II) have not been screened for possible anti­fungal activity. The X-ray crystal structure of bis­(phenyl­glycinato)palladium(II) containing two mol­ecules of dimethyl sulfoxide has been determined (Gao *et al.*, 2009 ▸). These authors also synthesized bis­(phenyl­glycinato)platinum(II), which also contains two mol­ecules of dimethyl sulfoxide, and showed that this platinum complex had a stronger binding affinity to fish-sperm DNA than the corres­ponding palladium complex. Both complexes added to DNA by a strong inter­calating mode and both complexes could cleave pBR 332plasmid DNA. (A plasmid is a small DNA mol­ecule within a cell that is physically separated from chromo­somal DNA and replicates independently. Plasmids are commonly found in bacteria as circular double-stranded DNA. Plasmid DNA can also be found in fungi and higher plants.) The palladium and platinum complexes are also cytotoxic to HeLa, Hep-G2, KB, and AGZY-83a tumor cells, with the platinum complex being more effective than the palladium complex. X-ray crystal structures have been reported for the palladium(II) complexes of glycine with 2,2′-bi­pyridine, 1,10-phenathroline or 2,2′-bi­pyridyl­amine with chloride counter-ions (Yodoshi & Okabe, 2008 ▸). Each of the complexes was shown to be capable of inter­calative binding to calf thymus DNA and could enhance the cleavage of pBR 332 plasmid DNA in the presence of hydrogen peroxide and ascorbic acid (Yodoshi & Okabi, 2008) . Small mol­ecules can inter­calate DNA by fitting in between base pairs in the two different DNA strands. Generally these mol­ecules are planar or nearly planar. In the case of the palladium(II) complex with glycine and bi­pyridine, the central palladium(II) atom has a distorted square-planar geometry. Furthermore, the two five-membered rings formed by the bi­pyridine and the glycine ligands are almost planar and the two pyridine rings are planar (Yodoshi & Okabe, 2008 ▸).

## Synthesis and crystallization   

Poly{caesium [*cis*-di­chloro-(*S*-2-amino-3-hy­droxy­propano­ate-κ^2^ *N*,*O*)palladate(II)]} was synthesized by a previously described method (Charlson *et al.*, 1981 ▸). Using a procedure similar to the method described for the synthesis of potassium-l-alaninato-di­chloro­platinum(II) (Ley & Ficken, 1912 ▸), a crude amorphous sample of potassium *cis*-di­chloro-l-serinato palladium(II) was obtained. Therefore, the potassium salt was converted by a known method (Cleare, 1977 ▸) into crystalline caesium *cis*-di­chloro-l-serinato palladium(II), which could be purified by recrystallization from water. In a typical preparation, a solution of l-serine (2.1 g) and potassium tetra­chloro­palladate(II) (3.2 g) in water (60 mL) was heated for 3h under reflux on a boiling water bath. Absolute ethanol (450 mL) was added to the filtered reaction mixture and the light-orange precipitate (1.7 g) was filtered off. This potassium salt of the palladium l-serine complex was reprecipitated from water (10 mL) with ethanol (40 mL). Small qu­anti­ties of solid caesium chloride were added to a stirred solution of the potassium salt (1.5 g) in water (10 mL) until the solution became dark red. A brick-shaped red crystalline caesium salt (1.2 g) was obtained by keeping this solution for 24 h at 278 K. The caesium complex was purified by two recrystallizations from water (yield 0.2 g). Analysis found: C, 8.83; H, 1.55; Cl, 17.2; N, 3.43. Calculated for C_3_H_6_Cl_2_NO_3_PdCs: C, 8.70; H, 1.46; Cl, 17.1; N, 3.38%.

## Refinement   

Crystal data, data collection and structure refinement details are summarized in Table 4 ▸. All H atoms were positioned geometrically with *d*(N—H) = 0.91 Å, for C*sp* ^3^—H, *d*(C—H) = 0.99 Å and (O—H) = 0.87 Å and with *U*_iso_(H) = 1.2*U*_eq_(C,N) or 1.5*U*_eq_(O).

## Supplementary Material

Crystal structure: contains datablock(s) I. DOI: 10.1107/S2056989017016164/im2483sup1.cif

Structure factors: contains datablock(s) I. DOI: 10.1107/S2056989017016164/im2483Isup2.hkl

Table 2. DOI: 10.1107/S2056989017016164/im2483sup3.pdf

CCDC reference: 1584689

Additional supporting information:  crystallographic information; 3D view; checkCIF report

## Figures and Tables

**Table 1:** Selected bond lengths (Å)

Cs1—Cs1^i^	4.421 (2)	Cs1—O2	3.117 (10)
Cs1—Pd1^ii^	3.8755 (17)	Cs1—O2^vi^	2.962 (10)
Cs1—Cl1^iii^	3.528 (4)	Cs1—O3	3.349 (10)
Cs1—Cl1^iv^	3.572 (4)	Cs1—C1	3.654 (15)
Cs1—Cl1^ii^	3.740 (4)	Pd1—Cl1	2.305 (4)
Cs1—Cl1^v^	3.698 (4)	Pd1—Cl2	2.287 (4)
Cs1—Cl2^ii^	3.510 (4)	Pd1—O1	1.993 (9)
Cs1—Cl2^v^	3.901 (4)	Pd1—N1	2.000 (12)

Symmetry codes: (i) 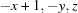; (ii) 
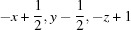
; (iii) 
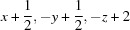
; (iv) 
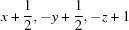
; (v) 
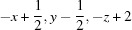
; (vi) 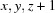.

**Table 2:** Hydrogen-bond geometry (Å, °)

*D*—H⋯*A*	*D*—H	H⋯*A*	*D*⋯*A*	*D*—H⋯*A*
O3—H3⋯O1^iv^	0.91 (1)	2.07 (2)	2.750 (14)	131 (3)
O3—H3⋯O2^iv^	0.91 (1)	2.60 (2)	3.479 (15)	163 (3)
N1—H1*A*⋯Cl2^vii^	0.91	2.62	3.471 (13)	156
N1—H1*B*⋯Cl2^viii^	0.91	2.51	3.388 (13)	163
C2—H2⋯O3^vii^	1.00	2.58	3.255 (19)	125

Symmetry codes: (iv) 
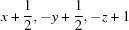
; (vii) 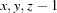; (viii) 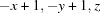.

**Table 3:** Mol­ecular geometry (Å, °) of the five-membered ring in *M*Cl_2_ (amino acid) complexes with Pt and Pd Data obtained from a search of the CSD (Groom *et al.*, 2016 ▸).

CCDC refcode	Reference	Structure	C—O	C=O	C—C	C—N	O—C—C—N (τ)
ACEMEC	Schiesser *et al.*, (2012 ▸)	K[Pt(L-alaO)Cl_2_]	1.304	1.223	1.528	1.480	25.85
GAWYOS	Bino *et al.*, (1988 ▸)	[PtCl_2_(*N*,*O*-Dap)]	1.313	1.232	1.543	1.499	19.44
GAWYUY	Bino *et al.*, (1988 ▸)	[PtCl_2_(*N*,*O*-Lys)]·H_2_O	1.300	1.219	1.500	1.557	13.66
GAWYPS	Bino *et al.*, (1988 ▸)	[PtCl_2_(*N*,*O*-Lys)]·H_2_O	1.315	1.227	1.457	1.436	15.72
KCGLPD	Baidina *et al.*, (1980*b* ▸)	K[Pd(Gly)Cl_2_]·H_2_O	1.285	1.216	1.518	1.490	11.74
BAGLPD	Baidina *et al.*, (1980*a* ▸)	Ba[Pd(Gly)Cl_2_]·2H_2_O	1.268	1.194	1.526	1.484	−13.69
BAGLPD	Baidina *et al.*, (1980*a* ▸)	Ba[Pd(Gly)Cl_2_]·2H_2_O	1.284	1.233	1.503	1.507	5.36

**Table 4:** Experimental details

Crystal data	
Chemical formula	[CsPd(C_3_H_6_NO_3_)Cl_2_]
*M*_r_	414.30
Crystal system, space group	Orthorhombic, *P*2_1_2_1_2
Temperature (K)	150
*a*, *b*, *c* (Å)	11.594 (4), 17.072 (5), 4.4739 (12)
*V* (Å^3^)	885.6 (5)
*Z*	4
Radiation type	Mo *K*α
μ (mm^−1^)	6.71
Crystal size (mm)	0.08 × 0.07 × 0.03

Data collection	
Diffractometer	Bruker APEXII CCD
Absorption correction	Multi-scan (*SADABS*; Bruker, 2016 ▸)
*T*_min_, *T*_max_	0.499, 0.746
No. of measured, independent and observed [*I* > 2σ(*I*)] reflections	5126, 1546, 1212
*R*_int_	0.125
(sin θ/λ)_max_ (Å^−1^)	0.595

Refinement	
*R*[*F* ^2^ > 2σ(*F* ^2^)], *wR*(*F* ^2^), *S*	0.039, 0.076, 0.83
No. of reflections	1546
No. of parameters	91
No. of restraints	13
H-atom treatment	H atoms treated by a mixture of independent and constrained refinement
Δρ_max_, Δρ_min_ (e Å^−3^)	1.64, −1.24
Absolute structure	Flack *x* determined using 375 quotients [(*I* ^+^)−(*I* ^−^)]/[(*I* ^+^)+(*I* ^−^)] (Parsons *et al.*, 2013 ▸)
Absolute structure parameter	−0.02 (5)

Computer programs: *APEX2* and *SAINT* (Bruker, 2016 ▸), *SHELXT* (Sheldrick, 2015*a* ▸), *SHELXL2014* (Sheldrick, 2015*b* ▸) and *OLEX2* (Dolomanov *et al.*, 2009 ▸).

## supplementary crystallographic information

### Crystal data

**Table:**

[CsPd(C_3_H_6_NO_3_)Cl_2_]	*D*_x_ = 3.107 Mg m^−^^3^
*M**_r_* = 414.30	Mo *K*α radiation, λ = 0.71073 Å
Orthorhombic, *P*2_1_2_1_2	Cell parameters from 360 reflections
*a* = 11.594 (4) Å	θ = 2.4–20.1°
*b* = 17.072 (5) Å	µ = 6.71 mm^−^^1^
*c* = 4.4739 (12) Å	*T* = 150 K
*V* = 885.6 (5) Å^3^	Plate, light yellow
*Z* = 4	0.08 × 0.07 × 0.03 mm
*F*(000) = 760	

### Data collection

**Table:**

Bruker APEXII CCD diffractometer	1212 reflections with *I* > 2σ(*I*)
φ and ω scans	*R*_int_ = 0.125
Absorption correction: multi-scan (SADABS; Bruker, 2016)	θ_max_ = 25.0°, θ_min_ = 2.1°
*T*_min_ = 0.499, *T*_max_ = 0.746	*h* = −13→13
5126 measured reflections	*k* = −20→16
1546 independent reflections	*l* = −5→3

### Refinement

**Table:**

Refinement on *F*^2^	Hydrogen site location: mixed
Least-squares matrix: full	H atoms treated by a mixture of independent and constrained refinement
*R*[*F*^2^ > 2σ(*F*^2^)] = 0.039	*w* = 1/[σ^2^(*F*_o_^2^)] where *P* = (*F*_o_^2^ + 2*F*_c_^2^)/3
*wR*(*F*^2^) = 0.076	(Δ/σ)_max_ = 0.001
*S* = 0.83	Δρ_max_ = 1.64 e Å^−^^3^
1546 reflections	Δρ_min_ = −1.24 e Å^−^^3^
91 parameters	Absolute structure: Flack *x* determined using 375 quotients [(*I*^+^)-(*I*^-^)]/[(*I*^+^)+(*I*^-^)] (Parsons *et al.*, 2013)
13 restraints	Absolute structure parameter: −0.02 (5)

### Special details

**Table:**

Geometry. All esds (except the esd in the dihedral angle between two l.s. planes) are estimated using the full covariance matrix. The cell esds are taken into account individually in the estimation of esds in distances, angles and torsion angles; correlations between esds in cell parameters are only used when they are defined by crystal symmetry. An approximate (isotropic) treatment of cell esds is used for estimating esds involving l.s. planes.

### Fractional atomic coordinates and isotropic or equivalent isotropic displacement parameters (Å^2^)

**Table:**

	*x*	*y*	*z*	*U*_iso_*/*U*_eq_	
Cs1	0.38524 (10)	0.10339 (5)	0.6704 (2)	0.0179 (3)	
Pd1	0.28780 (11)	0.41984 (6)	0.5719 (2)	0.0097 (3)	
Cl1	0.1180 (4)	0.4294 (2)	0.8375 (9)	0.0184 (9)	
Cl2	0.3493 (4)	0.5375 (2)	0.7574 (8)	0.0164 (10)	
O1	0.2471 (9)	0.3141 (6)	0.415 (2)	0.016 (3)	
O2	0.3137 (9)	0.2136 (6)	0.147 (2)	0.020 (3)	
O3	0.5412 (9)	0.2649 (6)	0.538 (2)	0.016 (2)	
H3	0.6175 (15)	0.2695 (16)	0.579 (8)	0.024*	
N1	0.4279 (10)	0.4068 (7)	0.316 (3)	0.014 (2)	
H1A	0.430153	0.446008	0.178195	0.017*	
H1B	0.492081	0.410758	0.431882	0.017*	
C1	0.3228 (14)	0.2811 (9)	0.245 (3)	0.012 (4)	
C2	0.4286 (13)	0.3293 (8)	0.158 (4)	0.014 (2)	
H2	0.424782	0.339538	−0.061897	0.017*	
C3	0.5389 (14)	0.2847 (9)	0.222 (3)	0.016 (2)	
H3A	0.541636	0.236400	0.099494	0.019*	
H3B	0.606568	0.317387	0.170155	0.019*	

### Atomic displacement parameters (Å^2^)

**Table:**

	*U*^11^	*U*^22^	*U*^33^	*U*^12^	*U*^13^	*U*^23^
Cs1	0.0240 (7)	0.0163 (5)	0.0134 (5)	0.0002 (5)	−0.0022 (5)	−0.0020 (4)
Pd1	0.0094 (7)	0.0098 (6)	0.0097 (6)	0.0012 (6)	0.0003 (6)	0.0000 (5)
Cl1	0.015 (2)	0.0202 (19)	0.020 (2)	0.001 (2)	0.003 (2)	−0.0022 (17)
Cl2	0.016 (3)	0.0124 (19)	0.021 (3)	−0.0018 (19)	0.0006 (18)	−0.0039 (15)
O1	0.009 (6)	0.015 (5)	0.025 (7)	−0.003 (5)	0.011 (5)	−0.005 (5)
O2	0.024 (8)	0.013 (5)	0.024 (6)	−0.001 (5)	−0.010 (6)	−0.003 (5)
O3	0.010 (5)	0.022 (5)	0.016 (5)	0.002 (4)	0.000 (4)	0.002 (4)
N1	0.008 (5)	0.018 (5)	0.017 (6)	0.002 (4)	−0.004 (5)	−0.001 (5)
C1	0.008 (9)	0.021 (9)	0.007 (9)	−0.001 (7)	0.005 (6)	0.003 (6)
C2	0.008 (5)	0.018 (5)	0.017 (6)	0.002 (4)	−0.004 (5)	−0.001 (5)
C3	0.010 (5)	0.022 (5)	0.016 (5)	0.002 (4)	0.000 (4)	0.002 (4)

### Geometric parameters (Å, º)

**Table:**

Cs1—Cs1^i^	4.421 (2)	Pd1—O1	1.993 (9)
Cs1—Pd1^ii^	3.8755 (17)	Pd1—N1	2.000 (12)
Cs1—Cl1^iii^	3.528 (4)	O1—C1	1.291 (17)
Cs1—Cl1^iv^	3.572 (4)	O2—C1	1.238 (18)
Cs1—Cl1^ii^	3.740 (4)	O3—H3	0.907 (12)
Cs1—Cl1^v^	3.698 (4)	O3—C3	1.458 (18)
Cs1—Cl2^ii^	3.510 (4)	N1—H1A	0.9100
Cs1—Cl2^v^	3.901 (4)	N1—H1B	0.9100
Cs1—O2	3.117 (10)	N1—C2	1.500 (17)
Cs1—O2^vi^	2.962 (10)	C1—C2	1.53 (2)
Cs1—O3	3.349 (10)	C2—H2	1.0000
Cs1—C1	3.654 (15)	C2—C3	1.52 (2)
Pd1—Cl1	2.305 (4)	C3—H3A	0.9900
Pd1—Cl2	2.287 (4)	C3—H3B	0.9900
			
Pd1^ii^—Cs1—Cs1^i^	70.48 (4)	O3—Cs1—C1	48.1 (3)
Pd1^ii^—Cs1—Cl2^v^	65.76 (6)	C1—Cs1—Cs1^i^	141.5 (2)
Cl1^v^—Cs1—Cs1^i^	50.56 (7)	C1—Cs1—Pd1^ii^	115.0 (2)
Cl1^iii^—Cs1—Cs1^i^	54.04 (6)	C1—Cs1—Cl1^ii^	109.9 (2)
Cl1^iv^—Cs1—Cs1^i^	54.55 (6)	C1—Cs1—Cl1^v^	167.2 (3)
Cl1^ii^—Cs1—Cs1^i^	51.09 (7)	C1—Cs1—Cl2^v^	116.3 (3)
Cl1^iv^—Cs1—Pd1^ii^	94.97 (7)	Cl1—Pd1—Cs1^vii^	69.20 (10)
Cl1^iii^—Cs1—Pd1^ii^	116.25 (7)	Cl2—Pd1—Cs1^vii^	63.44 (11)
Cl1^ii^—Cs1—Pd1^ii^	35.18 (7)	Cl2—Pd1—Cl1	90.97 (14)
Cl1^v^—Cs1—Pd1^ii^	60.77 (7)	O1—Pd1—Cs1^vii^	120.7 (3)
Cl1^iii^—Cs1—Cl1^v^	60.58 (12)	O1—Pd1—Cl1	92.5 (3)
Cl1^iii^—Cs1—Cl1^iv^	78.11 (9)	O1—Pd1—Cl2	175.4 (3)
Cl1^iv^—Cs1—Cl1^v^	105.11 (6)	O1—Pd1—N1	83.7 (4)
Cl1^iii^—Cs1—Cl1^ii^	105.13 (6)	N1—Pd1—Cs1^vii^	110.5 (3)
Cl1^v^—Cs1—Cl1^ii^	73.95 (7)	N1—Pd1—Cl1	175.3 (4)
Cl1^iv^—Cs1—Cl1^ii^	59.79 (11)	N1—Pd1—Cl2	93.0 (3)
Cl1^iii^—Cs1—Cl2^v^	94.47 (10)	Cs1^viii^—Cl1—Cs1^ix^	119.79 (11)
Cl1^v^—Cs1—Cl2^v^	50.97 (8)	Cs1^x^—Cl1—Cs1^ix^	75.40 (8)
Cl1^ii^—Cs1—Cl2^v^	99.35 (9)	Cs1^ix^—Cl1—Cs1^vii^	73.95 (7)
Cl1^iv^—Cs1—Cl2^v^	154.03 (9)	Cs1^x^—Cl1—Cs1^vii^	119.82 (11)
Cl1^iii^—Cs1—C1	127.5 (3)	Cs1^viii^—Cl1—Cs1^vii^	74.36 (8)
Cl1^iv^—Cs1—C1	87.0 (3)	Cs1^x^—Cl1—Cs1^viii^	78.11 (9)
Cl2^v^—Cs1—Cs1^i^	100.91 (6)	Pd1—Cl1—Cs1^vii^	75.62 (10)
Cl2^ii^—Cs1—Cs1^i^	102.14 (7)	Pd1—Cl1—Cs1^viii^	107.83 (14)
Cl2^ii^—Cs1—Pd1^ii^	35.65 (6)	Pd1—Cl1—Cs1^x^	164.55 (15)
Cl2^ii^—Cs1—Cl1^iii^	151.89 (9)	Pd1—Cl1—Cs1^ix^	111.88 (14)
Cl2^ii^—Cs1—Cl1^v^	93.39 (9)	Cs1^vii^—Cl2—Cs1^ix^	74.06 (8)
Cl2^ii^—Cs1—Cl1^ii^	53.58 (9)	Pd1—Cl2—Cs1^ix^	105.91 (14)
Cl2^ii^—Cs1—Cl1^iv^	100.86 (10)	Pd1—Cl2—Cs1^vii^	80.92 (12)
Cl2^ii^—Cs1—Cl2^v^	74.06 (8)	C1—O1—Pd1	116.2 (9)
Cl2^ii^—Cs1—C1	80.1 (3)	Cs1^xi^—O2—Cs1	94.8 (3)
O2—Cs1—Cs1^i^	129.96 (19)	C1—O2—Cs1^xi^	145.3 (10)
O2^vi^—Cs1—Cs1^i^	132.5 (2)	C1—O2—Cs1	105.8 (9)
O2^vi^—Cs1—Pd1^ii^	124.7 (2)	Cs1—O3—H3	124.3 (19)
O2—Cs1—Pd1^ii^	98.06 (19)	C3—O3—Cs1	110.7 (8)
O2^vi^—Cs1—Cl1^iii^	82.3 (2)	C3—O3—H3	101 (2)
O2—Cs1—Cl1^ii^	91.2 (2)	Pd1—N1—H1A	109.2
O2—Cs1—Cl1^iv^	79.5 (2)	Pd1—N1—H1B	109.2
O2—Cs1—Cl1^v^	158.4 (2)	H1A—N1—H1B	107.9
O2^vi^—Cs1—Cl1^ii^	159.9 (2)	C2—N1—Pd1	111.9 (9)
O2—Cs1—Cl1^iii^	140.2 (2)	C2—N1—H1A	109.2
O2^vi^—Cs1—Cl1^v^	94.5 (2)	C2—N1—H1B	109.2
O2^vi^—Cs1—Cl1^iv^	140.3 (2)	O1—C1—Cs1	101.0 (8)
O2^vi^—Cs1—Cl2^ii^	112.3 (2)	O1—C1—C2	117.5 (13)
O2—Cs1—Cl2^v^	118.8 (2)	O2—C1—Cs1	55.1 (8)
O2—Cs1—Cl2^ii^	65.0 (2)	O2—C1—O1	123.8 (15)
O2^vi^—Cs1—Cl2^v^	61.0 (2)	O2—C1—C2	118.7 (14)
O2^vi^—Cs1—O2	94.8 (3)	C2—C1—Cs1	114.9 (9)
O2—Cs1—O3	60.9 (3)	N1—C2—C1	110.5 (13)
O2^vi^—Cs1—O3	75.8 (3)	N1—C2—H2	108.0
O2—Cs1—C1	19.0 (3)	N1—C2—C3	111.1 (12)
O2^vi^—Cs1—C1	78.0 (3)	C1—C2—H2	108.0
O3—Cs1—Cs1^i^	109.42 (18)	C3—C2—C1	111.0 (13)
O3—Cs1—Pd1^ii^	153.60 (18)	C3—C2—H2	108.0
O3—Cs1—Cl1^iii^	80.07 (19)	O3—C3—C2	108.4 (14)
O3—Cs1—Cl1^ii^	123.52 (19)	O3—C3—H3A	110.0
O3—Cs1—Cl1^iv^	66.99 (18)	O3—C3—H3B	110.0
O3—Cs1—Cl1^v^	140.51 (19)	C2—C3—H3A	110.0
O3—Cs1—Cl2^v^	136.84 (18)	C2—C3—H3B	110.0
O3—Cs1—Cl2^ii^	125.87 (19)	H3A—C3—H3B	108.4

Symmetry codes: (i) −*x*+1, −*y*, *z*; (ii) −*x*+1/2, *y*−1/2, −*z*+1; (iii) *x*+1/2, −*y*+1/2, −*z*+2; (iv) *x*+1/2, −*y*+1/2, −*z*+1; (v) −*x*+1/2, *y*−1/2, −*z*+2; (vi) *x*, *y*, *z*+1; (vii) −*x*+1/2, *y*+1/2, −*z*+1; (viii) *x*−1/2, −*y*+1/2, −*z*+1; (ix) −*x*+1/2, *y*+1/2, −*z*+2; (x) *x*−1/2, −*y*+1/2, −*z*+2; (xi) *x*, *y*, *z*−1.

### Hydrogen-bond geometry (Å, º)

**Table:**

*D*—H···*A*	*D*—H	H···*A*	*D*···*A*	*D*—H···*A*
O3—H3···O1^iv^	0.91 (1)	2.07 (2)	2.750 (14)	131 (3)
O3—H3···O2^iv^	0.91 (1)	2.60 (2)	3.479 (15)	163 (3)
N1—H1*A*···Cl2^xi^	0.91	2.62	3.471 (13)	156
N1—H1*B*···Cl2^xii^	0.91	2.51	3.388 (13)	163
C2—H2···O3^xi^	1.00	2.58	3.255 (19)	125

Symmetry codes: (iv) *x*+1/2, −*y*+1/2, −*z*+1; (xi) *x*, *y*, *z*−1; (xii) −*x*+1, −*y*+1, *z*.
