# Supplementary material for: Crystal structure of poly[[μ3-(S)-2-amino-3-hydroxy­propano­ato]-cis-di-μ-chlorido-caesium­palladium(II)]
Source: Acta Crystallogr E Crystallogr Commun. 2017 Nov 21;73(Pt 12):1898–902. doi: 10.1107/S2056989017016164 (PMC5730248; doi:10.1107/S2056989017016164)
Supplement: Supplementary file 3 [file e-73-01898-sup3.pdf]

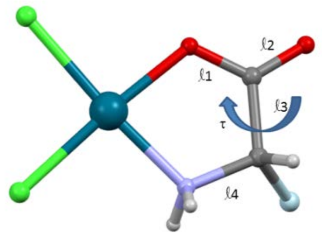

Table 2: Molecular geometry for planarity of 5-membered ring in MCl2 (amino acid) complexes with Pt and Pd

CCDC ref code	structure	l1	l2	l3	l4	torsion (τ)
ACEMEC	**K[Pt(L-alaO)Cl2]**	**1.304**	**1.223**	**1.528**	**1.480**	**25.85**
GAWYOS	**$[PtCl_2$(*N*,*O*-Dap)]**	**1.313**	**1.232**	**1.543**	**1.499**	**19.44**
**GAWYOS**	**$[PtCl_2$(*N*,*O*-Lys)]·H_20**	**1.300**	**1.219**	**1.500**	**1.557**	**13.66**
**GAWYPS**	**$[PtCl_2$(*N*,*O*-Lys)]·H_20**	**1.315**	**1.227**	**1.457**	**1.436**	**15.72**
**KCGLPD**	**K[Pd (Gly) Cl_2]·H_20**	**1.285**	**1.216**	**1.518**	**1.490**	**11.74**
**BAGLPD**	**Ba[Pd (Gly) Cl_2]·2H_20**	**1.268**	**1.194**	**1.526**	**1.484**	**-13.69**
**BAGLPD**	**Ba[Pd (Gly) Cl_2]·2H_20**	**1.284**	**1.233**	**1.503**	**1.507**	**5.36**
